# Oral Microbiome Dynamics in Treated Childhood Caries: A Comparative Study

**DOI:** 10.3390/life14121576

**Published:** 2024-12-01

**Authors:** Zahirrah Begam Mohamed Rasheed, Chew Wei Sheng, Erika Norfitriah, Nurrul Shaqinah Nasruddin, Farinawati Yazid

**Affiliations:** 1 Department of Craniofacial Diagnostics & Biosciences, Faculty of Dentistry, Universiti Kebangsaan Malaysia, Jalan Raja Muda Abdul Aziz, Kuala Lumpur 50300, Malaysia; zahirrah.rasheed@ukm.edu.my (Z.B.M.R.); p130597@siswa.ukm.edu.my (E.N.); shaqinah@ukm.edu.my (N.S.N.); 2 Department of Family Oral Health, Faculty of Dentistry, Universiti Kebangsaan Malaysia, Jalan Raja Muda Abdul Aziz, Kuala Lumpur 50300, Malaysia; p108041@siswa.ukm.edu.my

**Keywords:** oral microbiome, children’s oral health, dental caries, dmft/DMFT index, 16S rRNA sequencing, Malaysian children

## Abstract

Background: Dental caries is a multifactorial disease that results from interactions of susceptible host, cariogenic microorganisms, and fermentable carbohydrate sources. Our study explored oral microbiome shifts in children before and after dental treatment. Methods: Initial saliva samples were collected from caries free, moderate caries, and severe caries children based on the decayed, missing, and filled teeth index (DMFT/dmft) index. After three months of dental treatment, second saliva samples were gathered from the moderate and severe caries groups. The microbiota was analyzed by 16S rRNA gene-based high-throughput sequencing. Results: Most children with caries were between seven and eight years of age (40%), from middle-income group families (61%), highly educated parents (18% secondary level and 75% tertiary level) with good oral hygiene practices. There was a significant increase in alpha-diversity post-dental intervention. Firmicutes, Bacteroidota, and Proteobacteria were abundant across all samples. Post-treatment, Actinobacteria, and Firmicutes significantly decreased (*p* < 0.05) while Fusobacteria, Proteobacteria, Spirochaetota, and Synergistota significantly increased (*p* < 0.05). At genus level, a decreased trend was seen in *Streptococcus*, *Prevotella_7*, and *Rothia* and an increased trend was seen in *Fusobacterium*, *Neisseria*, *Haemophilus*, and *Leptotrichia*, but was not statistically significant. Conclusions: This study on Malaysian children highlights that dental caries are influenced by factors like age, socioeconomic status, and diet, with oral microbiome diversity increasing post-treatment, though some harmful bacteria persist, indicating a need for targeted oral health education and further research on probiotics’ role in caries prevention.

## 1. Introduction

Dental caries, or also known as tooth decay or cavities, is a non-communicable oral disease characterized by the progressive breakdown of tooth structure [1]. The prevalence of dental caries among children is lower in developed countries such as in the United States in six–eight-year-old [2], United Arab Emirates in five–15-year-old [3], Spain in six–12-year-old [4], and, in less developed countries such as India, in three–12-year-old children [5]. Despite the global reduction in caries prevalence in some developed regions, caries remains a significant public health concern, affecting 60–90% of school children and the majority of adults with varying ranges [6], thus imposing a significant health burden on healthcare systems and society. In Malaysia, despite caries prevalence reducing over the past decade, addressing caries prevalence is crucial, as outlined in the National Oral Health Plan for Malaysia 2011–2020 to achieve a goal of reaching a caries-free status by 2030 [7].

Dental caries is influenced by a multifaceted interplay of complex interaction of host, microbial, and environmental factors. Dietary habits high in sugary [8] and carbohydrate-rich [9] foods provide essential fuel for acid-producing bacteria in the oral cavity, thus promoting caries development. Saliva, on the other hand, acts as a natural buffer to neutralize bacterial acids, and its effectiveness depends on its flow rate, buffering capacity, and composition. Therefore, individuals with decreased saliva production or altered salivary composition are more susceptible to developing dental caries [10]. Additionally, socioeconomic factors, including access to dental care and preventive measures, can lower the risk of caries, but lower-income populations generally experience higher rates of this disease [11].

Oral microbiome dysbiosis has been recognized to play a pivotal role in dental caries. Dysbiosis state occurs when the balance of the commensal microbiome is disrupted, leading to changes in functional composition and metabolic activities [12]. Oral microbiome studies demonstrated a distinct profile between dental caries and caries-free children, with children with caries exhibiting higher levels of *Streptococcus mutans* and *Lactobacillus* spp., along with lower microbial diversity [13,14]. This shift underscores the need to investigate the changes in microbial diversity and specific bacterial taxa following dental interventions.

Most oral microbiome profiling studies among children with caries has focused on specific age groups [13,14,15,16,17,18,19], and only a few have compared severe early childhood caries to caries-free cases or examined populations with varying caries prevalence [15,16,19]. This points to a research gap in studying the oral microbiome in pre- and post-treatment childhood caries, specifically within the seven to 12 age range. This study aims to bridge this knowledge gap by examining the shifts in abundance and diversity of the oral microbiome among Malaysian children with varying caries statuses following dental treatment. Findings from this study may inform targeted strategies for caries prevention, potentially integrating microbiome modulation approaches, such as probiotic therapies or dietary modifications, to promote long-term oral health in children.

## 2. Materials and Methods

### 2.1. Participants and Sample Collection

This study is a prospective cohort study conducted during 2021–2023. Participants aged seven until 12 years old were recruited from the Faculty of Dentistry, University Kebangsaan Malaysia (UKM), Canselor Tuanku Muhriz Hospital (HCTM), and UKM Specialist Children’s Hospital (HPKK). The inclusion criteria for the participants include: (i) mixed dentition, (ii) no history of antibiotic, antifungal or steroid intake within three months prior to sampling, (iii) no evidence of oral abscess of candidiasis, and (iv) medically fit and well. Any non-Malaysian and children who have taken any antibiotic, antifungal, or steroid three months before and after treatment were excluded. The study was approved by the Human Research Ethic Committee, Centre for Research, and Instrumentation Management, UKM (reference number PPI/111/8/JEP-2021-643). Informed consent and assent forms were obtained from the legal guardians and the participants prior to the interview and sample collection.

In the first phase of the study, a total of 100 participating children were recruited and, upon dental examination, the participants were divided into three groups as follows: children caries free (CF) (*n* = 33), children with moderate caries with dmft/DMFT score ≤ 3 (MC) (*n* = 34), and children with severe caries with dmft/DMFT score ≥ 4 (SC) (*n* = 33). Demographic information was obtained from the legal guardians of the children and an unstimulated whole-saliva sample was collected from each of the children. Prior to sending the participants for a comprehensive dental treatment, the children were provided with standard level of oral hygiene and subsequently the children received comprehensive dental treatment tailored to their needs, either on the chairside or under general anesthesia. This treatment may involve various interventions, such as preventive treatments, restorations of restorable teeth, pulp treatment, and extractions of teeth with a poor prognosis. For the second phase, only 20 children completed their comprehensive dental treatment and fulfilled the three-months waiting period after completion of dental treatment. They were then examined, and saliva samples were again collected in which 10 samples were in the moderate caries after treatment (MCAT) group and 10 samples were in the severe caries after treatment (SCAT) group.

### 2.2. DNA Extraction and 16S rRNA Gene Amplicon Sequencing

DNA was extracted from the whole saliva and purified using the NucleoSpin Tissue Mini kit for DNA from cells and tissue (Macherey–Nagel, Düren, Germany) according to the manufacturer’s instructions. The extracted DNA from each group was pooled and prior to submission to the APICAL for amplification and sequenced, the DNA was separated into triplicates for each group.

Quality assessment of raw reads was done using fastqc files, after which primers and adaptors were removed using Cutadapt 3.5 [20]. Paired-end reads were processed and merged using DADA2 V1.18. Chimera screening and taxonomy assignment were done using the SILVA nr database V138.1 (updated 10 March 2021). For phylogenetic analysis (for unifrac), sequence alignment was done using MUSCLE 3.8 [21], followed with phylogenetic tree building using Fasttree2 [22].

Paired-end reads were first removed of sequence adaptors and low-quality reads using BBDuk of the BBTools package. Forward and reverse reads were merged using USEARCH v11.0.667. All sequences shorter than 150 bp or longer than 600 bp (sequenced on the MiSeq platform) were removed from downstream processing. Reads were then aligned with 16S rRNA (SILVA Release 132) or UNITE ITS database and inspected for chimeric errors using VSEARCH v2.6.2. Merged reads were clustered de novo into OTUs at 97% similarity using UPARSE v11.0.667; rare OTUs with less than two reads (doubleton), which are often spurious, were deleted from downstream processing. A single representative sequence from each OTU was randomly chosen, and Pynast [23] was used to align and construct a phylogenetic tree against the SILVA 132 16S rRNA database. Taxonomic assignment of OTU was achieved using QIIME V1.9.1 against the SILVA database 16S rRNA database (release 132). Operational taxonomic units (OTUs) are cluster of sequences that have a sequence identity above a given threshold, often at 97% [24].

### 2.3. Statistical Analyses

Demographic and socioeconomic data were keyed into Excel spreadsheet (Microsoft Excel version 2211) prior to transfer to Statistical Package for the Social Sciences (SPSS version 23). The main analysis was descriptive statistics, with inferential statistics used for comparison. For the inferential analysis, statistical significance was when p value was less than 0.05 (*p* < 0.05). Alpha-diversity matrices (observed species, Chao1, Shannon and Simpson) were also subjected for fitness to a normal distribution by Shapiro–Wilk test and followed with analysis of variance (ANOVA) using IBM SPSS (Version 21.0, IBM Corp., Chicago, IL, USA) to test for a significant difference between each group. Statistical significance was determined at *p* < 0.05. Beta diversity matrices were subjected to Principal Coordinates Analysis (PCoA) to test for dissimilarity patterns among bacteria from samples of different groups by using MicrobiomeAnalyst (https://www.microbiomeanalyst.ca/ (accessed on 7 August 2024)). To identify the differences between all the different groups and bacterial composition, heatmaps were performed for phyla and genus for each group by using MicrobiomeAnalyst (https://www.microbiomeanalyst.ca/ (accessed on 7 August 2024)) followed by ANOVA testing to find the significant difference at *p* < 0.05.

## 3. Results

### 3.1. Association of Demographic and Socioeconomic on Caries Status

The demographic and socioeconomic characteristics of the study participants are shown in Table 1. Male children comprise 54% of the proportions; however, it is not statistically significant associated with caries status and gender (*p* = 0.606). The mean age of the participants in this study was 9.3 + 1.74, with more children aged 11–12 years in the CF group while majority of 7–8-year-old children were in the SC group. This showed a significant association between caries status and age (*p* = 0.047). According to the race, there was no statistically significant association with caries status. Looking at the socioeconomic background, the majority of the MC and SC participants were from middle-income families with a significant association with caries status (*p* < 0.01). The same significant association was seen between caries status and parent’s education level (*p* < 0.01), since more than half of the parents had received tertiary level education.

### 3.2. Association of Oral Hygiene Practice, Snacking Frequency and Milk/Yogurt Consumption with Caries Status

The oral hygiene practices of the children were assessed, and the results are tabulated in Table 2. More than half of the children (72%) practice tooth brushing and this resulted in a statistically significant association with the status of caries (*p* < 0.0001). Despite the use of fluoridated toothpaste during tooth brushing, there was no significant association with the caries status. On the other hand, the percentage of regular dental checkups irrespective of caries status were almost similar between children with regular dental checkups (55%) and children with fewer visits to the dentist for regular dental checkups (45%), thus showing no association with caries status. Snacking (*p* < 0.01, Cramer’s V = 0.7219) and milk or yogurt consumption (*p* < 0.01, Cramer’s V = 0.6424), however, were significantly and highly associated with caries status.

### 3.3. Analysis of rRNA Sequencing Results

**Figure 1:**
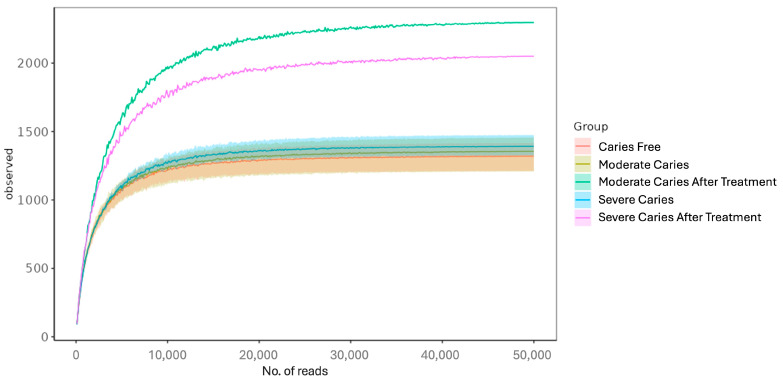
Rarefaction curve. The *x*-axis shows the number of valid sequences per sample and the *y*-axis shows the OTUs. Each curve in the graph represents different groups of samples and is shown in different colors. As the sequencing depth increases, the number of OTUs detected increases and the graph reaches plateau level at the end, indicating that most OTUs in the samples have been captured.

Illumina MiSeq sequencing platform was used to amplify and detect 16S rRNA gene product from oral microbiota of triplicate samples of CF, MC, SC, MCAT, and SCAT groups. The total number of reads after performing a series of processing steps was 1,647,500, with an average of 149,772 number of reads per sample. A total of 4531 operational taxonomic units (OTUs) were generated at a sequence-similarity level of 97%. The rarefaction curve for OTUs detected in this study showed that the quantity of observed OTUs increased as the sequencing depth increased. The ends of the rarefaction curves taper off, with increasing numbers of sequences per sample, as is commonly observed with sequencing data (Figure 1). A ribosomal database was used to classify sequences representing OTUs in which the bacteria detected were composed of 10 phyla, 15 class, 38 order, 66 family, and 125 genera.

### 3.4. Comparison of the Oral Microbiome in Children with Caries

**Figure 2:**
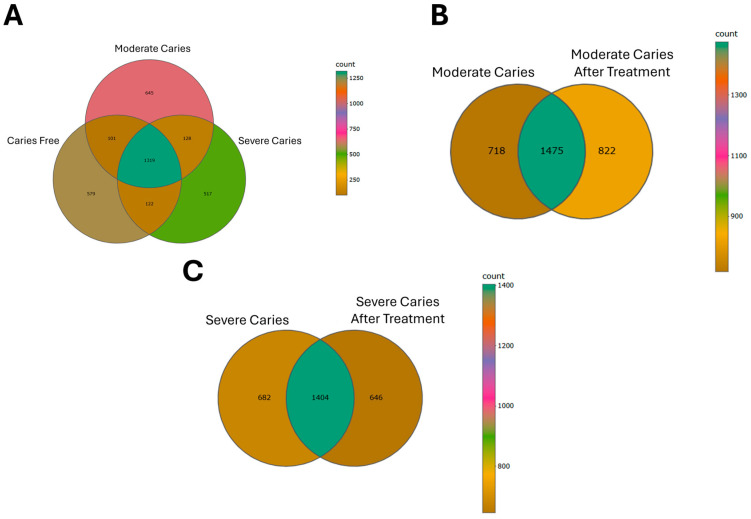
The Venn diagrams show the number of OTUs (97% sequence identity) that were shared or not shared by the different caries statuses before and after dental treatment, depending on the overlaps. (**A**) The number of OTUs shared by CF, MC, and SC. (**B**) The number of OTUs shared by MC and MCAT. (**C**) The number of OTUs shared by SC and SCAT.

Venn diagram was used to confirm the core oral microbiota present in the different groups of children with caries and after treatment in moderate caries and severe caries. The shared taxa by all individuals in each group were deemed to be the core bacterial communities. The number of OTUs common between CF, MC, and SC groups was 1319. After treatment, there were 1475 OTUs shared by MC and MCAT groups and 1404 by the SC and SCAT groups (Figure 2A–C).

**Figure 3:**
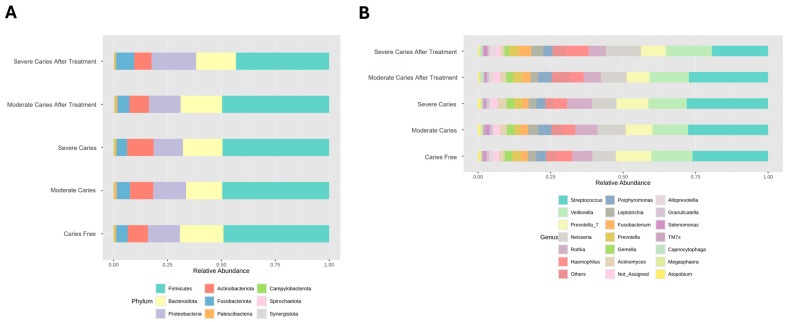
The bar charts show the relative abundance of the most dominant taxa at the (**A**) phyla level and the (**B**) genus level in CF, MC, SC, MCAT, and SCAT groups.

Analyzing taxonomic classification at the phylum level, CF, MC and SC groups were mainly constituted by Firmicutes (49.03%, 49.59%, 49.47%), Bacteroidota (20.20%, 16.77% and 18.34%), and Proteobacteria (14.83%, 15.22% and 13.60%), respectively (Figure 3A). After treatment, these three microbiotas remain almost the same in the MCAT group (Firmicutes 49.67%, Bacteroidota 19.27%, and Proteobacteria 14.68%) but a slight decrease was observed in the SCAT group in Firmicutes (43.23%) while a slight increase was seen in Proteobacteria (20.70%) (Figure 3A).

Whereas at the genus level, all three groups of children were represented by *Streptococcus* (26.06%, 27.62%, and 28.06%), *Veillonella* (14.27%, 12.28%, and 13.24%) and *Prevotella_7* (12.21%, 9.22%, and 11.00%), respectively with the exception of moderate caries whereby the third most abundant microbiota was *Neisseria* (9.70%). After treatment, in MC and SC groups, they were represented mainly by genus *Streptococcus* (27.35% and 19.39%), *Veillonella* (13.47% and 15.92%), and *Neisseria* (8.90% and 12.06%) (Figure 3B).

### 3.5. Diversity Analysis of Microbiota in Different Caries Statuses Before and After Dental Treatment

**Figure 4:**
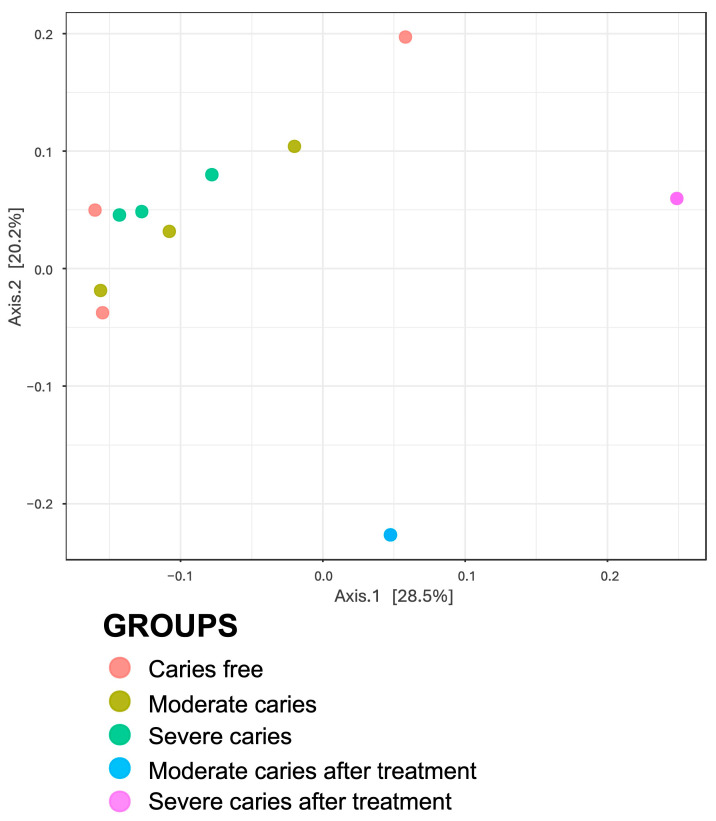
Principal Coordinate Analysis (PCoA) plot. Each sample group is represented by the same color. The percentage of the main coordinates represents the relative contribution of this coordinate to sample differences. The distances between the sample points represent the similarity of microbiota in the sample. A closer distance represents higher similarity and samples that cluster together are composed of similar microbiota.

The α-diversity and β-diversity indices of the different caries statuses were calculated before and after dental treatment. The α-diversity indices for Chao1, Shannon, and Simpson values varied between 1199.16–2410.30, 6.56–7.20, and 0.998–0.999, respectively, for individual samples (Appendix A, Table A1). In a group, the saliva samples from the SC group had higher Chao1 and Shannon compared to CF and MC; however, the diversity metrics showed no significant differences between these three groups (*p* > 0.05). Nonetheless, saliva samples from the MCAT group had significantly higher diversity in Chao1, Shannon, and Simpson compared to the MC group (*p* < 0.05). The same significant results were also obtained in the SCAT group compared to the SC group in Chao1, Shannon, and Simpson (*p* < 0.05) (Table 3). Principal Coordinates Analysis (PCoA) also demonstrated the differences of microbiota in the MCAT and SCAT samples compared to the MC and SV groups (Figure 4), in which the clustering of these samples is clearly separated.

### 3.6. Profiling the Effects of Dental Intervention on the Differences of Oral Microbiota in Children with Caries

**Figure 5:**
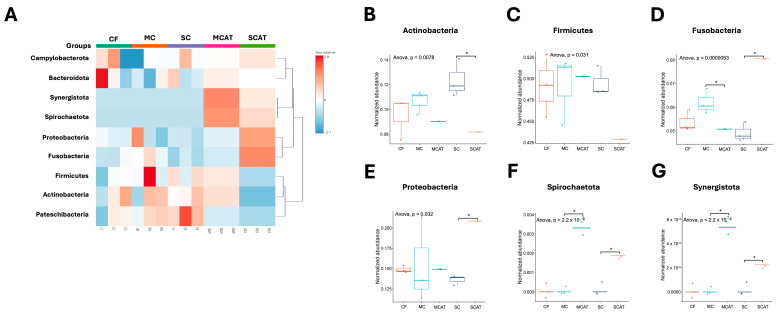
Hierarchical clustering of microbiota data at bacterial phyla level and differences in relative abundance (mean % + SD). (**A**) Microbiota abundances are color-coded according to the color key on the right side. Euclidean distance was used to cluster the rows and columns of the heatmap. The color bar on top of the heatmap is colored according to the sample group. The differences of the relative abundances of the major phyla after treatment. The significance of (**B**) Actinobacteria, (**C**) Firmicutes, (**D**) Fusobacteria, (**E**) Proteobacteria, (**F**) Spirochaetota, and (**G**) Synergistota were determined using the ANOVA test. * *p* < 0.05.

**Figure 6:**
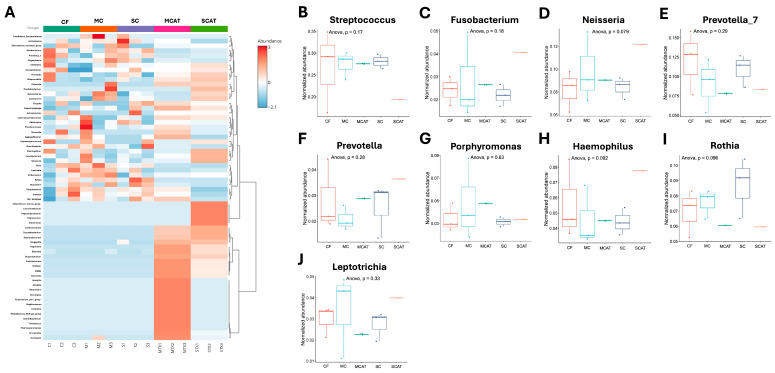
Hierarchical clustering of microbiota data at bacterial genus level and differences in relative abundance (mean % + SD). (**A**) Microbiota abundances are color-coded according to the color key on the right side. Euclidean distance was used to cluster the rows and columns of the heatmap. The color bar on top of the heatmap is colored according to the sample group. The differences of the relative abundances of major genus (**B**) *Streptococcus*, (**C**) *Fusobacterium*, (**D**) *Neisseria*, (**E**) *Prevotella_7*, (**F**) *Prevotella*, (**G**) *Porphyromonas*, (**H**) *Haemophilus*, (**I**) *Rothia*, and (**J**) *Leptotrichia* were determined using the ANOVA test. *Leptotrichia, Porphyromonas, Rothia, Haemophilus*—associated with caries free; *Neisseria, Streptococcus, Prevotella, Prevotella_7, Fusobacterium*—associated with caries active.

The differential bacterial taxon abundance in the different caries group microbiota datasets may be demonstrated by grouping based on the similarity of the taxa represented by vertical and horizontal dendograms. The relatedness levels between the samples are shown using a heatmap color scale to show the abundance levels as depicted in Figure 5 at a phyla level. There is clustering of MCAT and SCAT in this branch, showing a considerable similarity between these groups compared to the other groups at phyla level. The microbiotas of the MCAT and SCAT samples were characterized by the high relative abundance of Synergistota and Spirochaetota, in which these microbiotas are lowly expressed in the other caries groups. Proteobacteria and Fusobacteria were highly abundant in the SCAT group but were lowly abundant in the MCAT group, while Firmicutes and Actinobacteria were highly abundant in the MCAT but lowly abundant in the SCAT group (Figure 5A). Comparing these microbiotas after treatment, Actinobacteria significantly increased in the SCAT group (Figure 5B) while, although non-significant, there is a decrease trend of Firmicutes in the SCAT group (Figure 5C). Fusobacteria on the other hand showed a significant decrease in the MCAT group, while it significantly increased in the SCAT group (Figure 5D). A similar significant increase in the SCAT group is also observed in Proteobacteria (Figure 5E). Interestingly, both Spirochaetota (Figure 5F) and Synergistota (Figure 5G) showed significant increase in the MCAT and SCAT groups. At genus level, the MCAT and SCAT groups showed a distinct cluster compared to the other groups but the differences among these genera were not significant (Figure 6).

## 4. Discussion

The etiological concept of dental caries is multifactorial with the interplay of various factors including the host, the microorganisms, and diet. To our knowledge, this is the first study that gives insight into the microbial analysis of various stages of dental caries among children in Malaysia before and after dental treatment. The findings of this study highlight that the etiology of dental caries is a complex process, and it is incorrect to assume that it is identical in children and adults [25].

The susceptibility of an individual to dental caries is largely affected by the host factors’ demographic. In the pediatric population, research on gender disparities in dental caries showed inconclusive evidence with mixed results [26] in contrast to the extensive research among adolescences and adults that has consistently indicated that females bear a greater burden compared to males [27,28]. Although the significant relationship is missing in this study, females outnumbered males in the severe dental caries categories. The hormonal changes, different salivary composition and flow rate, and earlier eruption of teeth may be the reasons pointing to females as more susceptible [27]. The disparities in caries experience also vary with age, showing the younger age group have higher caries prevalence [29,30], aligned with the higher severe caries group among seven–eight-year-age group in this study. This supports the idea that dental health is worse at younger ages, since these children are mostly highly dependent on the parents for oral health. Remissness on the children’s oral health from the parents may stem from the time constraints of working on multiple jobs to supplement their primary income due to high financial stress, especially among the middle-class families [31,32]. Since more than half of the parents are in this group, this may explain the children’s behavior seen in this study. Nonetheless, parents with higher education levels generally exhibit better oral health knowledge and practices [33]. This behavior is translated in this study, with a majority of the children practice frequent toothbrushing and use fluoridated toothpaste. However, caries prevalence is still high in this group of children despite the use of fluoridated toothpaste. One significant factor that can contribute to this scenario is the frequent snacking, which exposes the teeth to a prolonged acidic state, thus promoting demineralization of the enamel [34]. Meanwhile, consumption of milk and yogurt play a protective role against tooth decay, primarily due to their nutritional composition and the presence of specific components that benefit oral health [35], as seen in this study. That component was specified by other researchers as minerals, proteins, and bioactive peptides that help remineralize carious tissue and inhibit the growth of caries-causing pathogens [36].

Previously, dental caries was associated with the single pathogen theory approach, but this was later refuted by a microecological imbalance theory, whereby the disease is viewed as the result of an imbalance in the oral microbiome rather than by specific pathogens alone [37]. Through a high-throughput 16S rRNA sequencing, the alpha diversity of the oral microbiome in the MC and SC groups was higher compared to CF group, but not statistically significant (Table 3). This may be due to the good homogeneity of the population that have similar living environments, cultural customs, diet, and lifestyles. A meta-analysis also reported a comparable finding that reported no significant difference in alpha diversity between caries and no caries [38]. Post-dental treatment, however, demonstrated a significant increase of richness and diversity compared to the MC and SC groups, suggesting the potential impact of treatment on the oral microbiome community. An acidic environment that favors aciduric bacteria is common in dental caries, in which after treatment, the pH of the oral environment may normalize thus reducing the dominance of acid-tolerant species and potentially contributing to a more diverse and even community structure [37]. Although the diversity seen after treatment may suggest a positive response to interventions, it is essential to further explore the specific taxonomic changes underlying these diversity shifts. Additionally, understanding the functional implications of increased diversity is crucial for assessing the overall health of the oral microbiome.

In this study, Firmicutes, Bacteroidota, and Proteobacteria were abundant at the phyla level across all groups, which is consistent with previous observations [39,40,41,42], thus manifesting the link of the roles of these microbes in oral health and disease [43] due to their consistent representation. The abundance of these microbiota remains roughly the same in the moderate caries group before and after treatment, proving a dysbiosis state in the oral microbiome with a high risk of caries recurrence even after treatment [44]. Several studies that indicated a significant reduction of cariogenic bacteria after caries dental treatment demonstrated a high and rapid relapse rate [45,46]. This could imply that these bacteria may have the ability to rapidly recolonize the oral surface after clinical interventions, due to the capacity to colonize multiple niches of the oral cavity, including the soft tissues [47]. Therefore, controlling the maturation of certain bacterial species is of the utmost important to improve oral microbiome and reduce the risk of disease recurrence.

Notable differences at the phyla level and genus level can be observed after treatment in the severe caries group, highlighting the potential impact of interventions on the oral microbiota landscape. Actinobacteria are a complex community of bacteria that adhere to tooth surfaces and contribute to the formation of biofilm and carbohydrate fermentation. The core existence of this bacteria creates an acidic environment, further paving a way for the pathogenic Firmicutes to colonize and thrive (reviewed in [37]). Reduced population of these bacteria after dental treatment may be indicated by the mechanical disruption by restorative procedures. Mechanical removal of carious tissues and biofilms that harbor high levels of these cariogenic bacteria and the source of nutrients for carbohydrate fermentation resulted in a reduction in the bacterial population [48]. A restoration to a more neutral pH environment in the oral cavity may also inhibit the thriving survival of these acidogenic bacteria [49]. Reduction of these bacteria has left a void in the ecological niches in the oral cavity and, as a compensatory mechanism, other microbes may increase in abundance. Evidence from other studies demonstrated the same observation with increased Fusobacteria in oral cavity after healthy food consumption [50] and Proteobacteria in a reshaping mechanism of a normal gut microbiome [51] with the reduction of Actinobacteria and Firmicutes. Thus, the increase of these bacteria in this study could indicate a normal nutrient cycling in an attempt to restore the normal oral-cavity environment. Although they are typically considered commensal bacteria that contribute positively to oral health, an overgrowth after treatment may suggest dysbiosis-induced and is likely to represent a transitional stage in the shift from healthy to diseased state [52]. This notion is concurrent with the increased in abundance of Synergistota and Spirochaetota, the main pathogens associated with periodontal disease [53,54], or the increase could also be an act of antagonism between caries and periodontal disease [55]. Progressed caries that penetrate deep into the tissue may favor the survival of these bacteria that thrive in low oxygen level environment. Their community may also increase with the adaptation to the changing environment following treatment with the metabolic capabilities to utilize metabolic byproducts of other bacteria, such as benzoate or lactate as an energy source, to reproduce [51,56] and maintain their community structure. However, further investigation is warranted to discern whether these changes are indicative of a restoration toward a healthier state or represent a microbial community adapted to post-treatment conditions or establishment of a disease-state microbiome.

Oral microbiome is also influenced by the intake of probiotics. Sources of probiotics that were investigated in this study showed that the consumption of milk and yogurt showed a strong correlation of reduced severe dental caries incidence among the children. *Streptococcus* plays a crucial role in oral health, especially in the contribution for the formation of dental biofilms and carbohydrate metabolism [57]. The decrease in this study is aligned with a study that shows different microbiome profiles with inverse association between milk intake and *Streptococcus mutans* colonization in individuals who frequently consumed milk in their diet [16], possibly via blocking the adhesion and metabolism of the *Streptococcus mutans* [19,40]. Although some microbiological changes were seen, the high diversity of microbiome, especially in anaerobic bacteria is seen in this study as compared to a reduction of anaerobic bacteria with Lactobacillus probiotic on murine models [58]. Therefore, a long-term change in oral microbiome composition and a detailed analysis of the shifts mediated by probiotics in humans warrant further investigation.

## 5. Limitation

The limitation of this study is the small sample size for each caries group, the pool samples after dental treatment for 16S rRNA sequencing, and a short recall period of three months after dental intervention in Phase II. Increasing the sample size and analyzing individual samples after treatment could enable a more in-depth analysis of the microbiome. Tracking the oral microbiome over extended periods provides a more comprehensive understanding of changes to the oral microbiome as more stable and long-lasting changes in the community structure and functional composition develop over extended periods of time. Thus, recommendations for future research are to use a larger sample size with a longer recall period with individual sequencing to obtain a more accurate and reliable result. The next limitations of this study would be the limited dietary information and limited geographic coverage during sampling. Detailed dietary information is crucial, as diet plays a significant role in shaping the composition and dynamics of the oral microbiome and, through extending the geographic coverage to other countries, would allow better understanding of other factors that could influence the abundance and diversity of oral microbiome such as local culture, living environment, cultural customs, diet, and lifestyles.

## 6. Conclusions

This study highlights several factors contributing to dental caries in children in the Malaysian population, particularly how the oral microbiomes change pre- and post-treatment. Our results show that the causes of dental caries are multi-dimensional, and factors like age and socioeconomic status may play a role in caries prevalence, suggesting that targeted oral health education for parents, especially in the middle-income families, is essential.

After dental treatment, we observed an increase in oral microbiome diversity, which provided a positive sign. However, the presence of certain harmful bacteria after dental treatment suggests that some children remain at risk for future caries. Additionally, we noted that diets rich in milk and yogurt were linked to lower caries rates, pointing to dietary choices rich in probiotics, and probiotic dental products, including toothpaste and mouthwash, as an important factor in modifying the microbial environment toward healthy oral microbiome.

In summary, further research is needed to discern the oral microbiome diversity and its implication in oral health, particularly in preventing dental caries among children.

## Figures and Tables

**Table 1:** Demographic and socioeconomic characteristics.

	Caries Free (N)	Moderate Caries (N)	Severe Caries (N)	Total N (%)	Chi-Square Test/* Fisher EXACT Test
Gender					
Male	20	18	16	54 (54%)	X2(2) = 1, *p* = 0.606
Female	13	16	17	46 (46%)	Cramer’s V = 0.1
Age					
7 to 8	8	16	16	40 (40%)	
9 to 10	10	13	8	31 (31%)	
11 to 12	15	5	9	29 (29%)	
Race					
Malay	24	27	28	79 (79%)	Fischer’s Exact Test, *p* = 0.646
Chinese	5	4	3	12 (12%)	Cramer’s V = 0.152
Indian	4	3	1	8 (8%)	
Other	0	0	1	1 (1%)	
Monthly Household Income					
B40 (Low)	3	6	7	16 (16%)	X2(2) = 27.87, *p* < 0.01
M40 (Middle)	12	25	24	61 (61%)	Cramer’s V = 0.373
T20 (High)	18	3	2	23 (23%)	
Parent’s Education Level					
Primary	0	0	7	7 (7%)	Fischer’s Exact Test, *p* < 0.0001
Secondary	3	5	10	18 (18%)	Cramer’s V = 0.342
Tertiary	30	19	16	75 (75%)	

* Fischer’s Exact Test was used when more than 20% of cells have expected frequencies <5.

**Table 2:** Oral Hygiene Practice, Snacking Frequency and Yogurt Consumption.

	Caries Free (N)	Moderate Caries (N)	Severe Caries (N)	Total N (%)	Chi-Square Test/* Fisher Exact Test
Tooth Brushing					
<2 times a day	0	9	19	28 (28%)	X2(2) = 27.19, *p* < 0.0001
≥2 times a day	33	25	14	72 (72%)	Cramer’s V = 0.521
Fluoridated Toothpaste					
Yes	32	34	33	99 (99%)	Fischer’s Exact Test, *p* = 0.660
No	1	0	0	1 (1%)	Cramer’s V = 0.143
Regular Dental Checkup					
Yes	21	21	13	55 (55%)	X2(2) = 4.87, *p* = 0.087
No	12	13	20	45 (45%)	Cramer’s V = 0.221
Snacking					
<3 times a day	31	24	3	58 (58%)	X2(2) = 52.11, *p* <0.01
≥3 times a day	2	10	30	42 (42%)	Cramer’s V = 0.7219
Milk/Yogurt Consumption					
Yes	29	23	4	56 (56%)	X2(2) = 41.27, *p* <0.01
No	4	11	29	44 (44%)	Cramer’s V = 0.6424

* Fischer’s Exact Test was used when more than 20% of cells have expected frequencies <5.

**Table 3:** Comparison of alpha-diversity indices of oral microbiota between the caries free (CF), moderate caries (MC), and severe caries (SC) groups before and after dental treatment.

	Within Groups						ANOVA with Post Hoc Test Between Groups				
Alpha Diversity	Caries Free	Moderate Caries	Severe Caries	Moderate Caries After Treatment	Severe Caries After Treatment	ANOVA	Caries Free vs. Moderate Caries	Caries Free vs. Severe Caries	Moderate Caries vs. Severe Caries	Moderate Caries vs. Moderate Caries After Treatment	Severe Caries vs. Severe Caries After Treatment
Observed species	1319.67 ± 107.62	1354.67 ± 138.15	1392.00 ± 79.54	2297 ± 22	2050 ± 30	F = 103.03, *p* < 0.001	*p* > 0.05	*p* > 0.05	*p* > 0.05	*p* = 0.0091	*p* = 0.0027
Chao1	1324.03 ±109.12	1360.21 ± 142.31	1396.25 ± 78.015	2315.55 ± 94.75	2074.44 ± 105.68	F = 102.63, *p* < 0.001	*p* > 0.05	*p* > 0.05	*p* > 0.05	*p* = 0.0092	*p* = 0.0025
Shannon	6.68 ±0.11	6.71 ± 0.12	6.72 ± 0.02	7.18 ± 0.02	7.16 ± 0.01	F = 35.51, *p* < 0.001	*p* > 0.05	*p* > 0.05	*p* > 0.05	*p* = 0.03	*p* < 0.0001
Simpson	0.998 ± 0.00	0.998 ± 0.00	0.998 ± 0.00	0.999 ± 0.00	0.999 ± 0.00	F = 8.452, *p* < 0.05	*p* > 0.05	*p* > 0.05	*p* > 0.05	*p* = 0.01	*p* = 0.0059

Values are shown as mean ± standard deviation. The Chao1 index was used to estimate the number of operational taxonomic units (OTUs) in samples and is commonly used in ecology to estimate the total number of species. The Shannon index and Simpson’s diversity index are common measures of diversity, which reflect richness and evenness of the samples.

## Data Availability

The data presented in this study are available on request from the corresponding author.

## Appendix A

**Table A1:** The α-diversity indices for individual samples.

Sample Name	Observed Species	Chao1	Shannon	Simpson
C1	1333	1341.26	6.78	0.998
C2	1206	1207.31	6.56	0.998
C3	1420	1423.50	6.69	0.998
M1	1198	1199.16	6.59	0.998
M2	1459	1469.03	6.70	0.998
M3	1407	1412.44	6.83	0.998
MTX1	2275	2306.25	7.18	0.999
MTX2	2297	2410.30	7.20	0.999
MTX3	2319	2230.10	7.16	0.999
S1	1479	1481.29	6.72	0.998
S2	1323	1328.00	6.70	0.998
S3	1374	1379.45	6.74	0.998
STX1	2000	2010.10	7.16	0.999
STX2	2080	2180.12	7.15	0.999
STX3	2070	2033.10	7.17	0.999
